# Preclinical Assessment of HIV Vaccines and Microbicides by Repeated Low-Dose Virus Challenges

**DOI:** 10.1371/journal.pmed.0020249

**Published:** 2005-07-19

**Authors:** Roland R Regoes, Ira M Longini, Mark B Feinberg, Silvija I Staprans

**Affiliations:** **1**Department of Biology, Emory UniversityAtlanta, GeorgiaUnited States of America; **2**Department of Biostatistics, Rollins School of Public Health, Emory UniversityAtlanta, GeorgiaUnited States of America; **3**Departments of Medicine and Microbiology and Immunology, Emory University School of Medicine and the Emory Vaccine CenterAtlanta, GeorgiaUnited States of America; **4**Merck Vaccine DivisionMerck, West Point, PennsylvaniaUnited States of America; Harvard School of Public HealthUnited States of America

## Abstract

**Background:**

Trials in macaque models play an essential role in the evaluation of biomedical interventions that aim to prevent HIV infection, such as vaccines, microbicides, and systemic chemoprophylaxis. These trials are usually conducted with very high virus challenge doses that result in infection with certainty. However, these high challenge doses do not realistically reflect the low probability of HIV transmission in humans, and thus may rule out preventive interventions that could protect against “real life” exposures. The belief that experiments involving realistically low challenge doses require large numbers of animals has so far prevented the development of alternatives to using high challenge doses.

**Methods and Findings:**

Using statistical power analysis, we investigate how many animals would be needed to conduct preclinical trials using low virus challenge doses. We show that experimental designs in which animals are repeatedly challenged with low doses do not require unfeasibly large numbers of animals to assess vaccine or microbicide success.

**Conclusion:**

Preclinical trials using repeated low-dose challenges represent a promising alternative approach to identify potential preventive interventions.

## Abstract 2

Trials of HIV vaccines in animals using repeated low- dose challenges of the virus are feasible and may be more true to life.

## Introduction

Worldwide approximately 40 million people are infected with HIV, and more than 3 million people died of AIDS last year alone [1]. Unfortunately, numerous obstacles to providing effective antiretroviral treatment to the majority of infected individuals in resource-poor countries exist. The development of a vaccine or other preventive biomedical intervention therefore bears the greatest hope to curb the rampant HIV epidemic [2].

Research on HIV vaccines and prevention relies strongly on preclinical studies in macaque models for the identification and evaluation of potential vaccines or prophylactic treatment strategies [3]. Initially, the goal was to use animal trials to screen for preventive interventions that induce sterilizing immunity (i.e., protection against infection) since this would clearly be the most effective way to contain the AIDS pandemic. Unfortunately, most of the vaccine approaches assessed to date in animal studies have failed to induce sterilizing immunity [4–7], although some prophylactic approaches were found to reduce susceptibility to infection [8–12]. As a result of this shortcoming, vaccine candidates are at present primarily examined with regard to their effects on set point viremia, disease progression, and their general immunogenicity, rather than with regard to the degree of protection against infection they confer. However, the inference as to the degree of sterilizing immunity from the level of immunogenicity is limited by our lack of knowledge about the mechanisms of protection against infection as such [13].

The inability of most vaccine candidates to induce protection against infection in animal studies may be due, at least in part, to unintended consequences of the design of the animal trials, rather than to problems inherent in the vaccination approaches themselves. In most animal studies that seek to test the efficacy of a given preventive intervention, very high challenge doses are used, typically of approximately 10–100 times the infectious dose at which 50% of the animals become infected (*ID*_50_). The motivation for using such high challenge doses is mostly practical: the experimenter wants to ascertain infection success in unvaccinated/untreated animals, which can then be compared to the hopefully lower infection success in vaccinated/treated animals. There are, however, concerns with using high challenge doses. Firstly, the extremely high probability of infection in high-dose challenge studies conflicts with the low transmission rate of HIV per sex act [14–17]. Although it has been argued that transmission rates may be higher under some circumstances (such as during acute infection or when other infections of the genital tract are present) than the estimates obtained from discordant couple studies suggest (e.g., the recent study by Pilcher et al. [18]), transmission of HIV during one sex act surely does not occur with certainty. Secondly, protection against high-dose virus challenges may be more difficult to achieve because the use of high challenge doses makes stochastic extinctions that can play an important role in early control of the infection [19] very unlikely. Thus, standard high-dose challenge studies may rule out preventive intervention strategies that could protect against infections following “real life” exposures.

The problems of using high virus doses in animal studies can be illustrated by the discrepancy between the protection zidovudine (AZT) confers in animals and humans. Whereas macaques [20,21] and mice [22] were not protected from infection with high challenge doses by zidovudine (a relatively weak antiretroviral drug when used in monotherapy), clinical studies surprisingly showed that two-thirds of perinatal infections (i.e., mother-to-child transmissions during birth) can be prevented by zidovudine administration [23]. It is important to note that the use of zidovudine to prevent perinatal HIV infection is a biomedical intervention aiming to protect from infection, whereas zidovudine is most commonly used as a therapeutic agent after infection. This example suggests that there is a need for experimental designs that allow the assessment of the protection against infection with lower, and thus more realistic, challenge doses.

**Figure 1:**
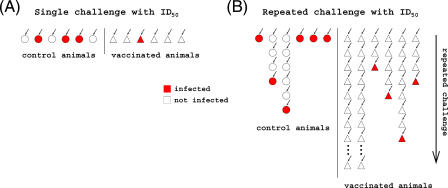
Single and Repeated Low-Dose Challenge Designs Figure shows designs for single (A) and repeated (B) low-dose challenge designs. Small arrows denote challenges, and white and red symbols denote uninfected and infected animals, respectively.

The belief that experiments involving realistically low challenge doses require unfeasibly large numbers of animals has prevented the development of low-dose challenge models. In this theoretical study, we show that, contrary to this widely held belief, low-dose challenge experiments can be designed such that they do not require large numbers of animals. Using statistical power analysis, we compare two experimental designs (see Figure 1): (i) a single low-dose challenge design in which each animal is challenged only once, and (ii) a repeated low-dose challenge design in which each animal is challenged until it is infected or a predetermined maximum number of challenges is reached. We find that the repeated low-dose challenge design does not require unfeasibly large numbers of animals.

In the following, we are going to discuss the case of assessing whether a vaccine candidate induces sterilizing immunity. All the considerations in this article, however, apply equally to other preventive interventions, such as microbicides.

## Methods

To assess the quality of the single and the repeated low-dose challenge designs, we conducted a statistical power analysis. The statistical power of an experimental design is defined as the probability that an effective vaccine or treatment is correctly determined to be effective. This analysis consists of simulating the experiments, evaluating them, and then repeating this procedure thousands of times to estimate the statistical power of a given experimental design.

### Simulation of Single Low-Dose Challenge Experiments

In our simulations of the single low-dose challenge experiments, we assume that we have *n* unvaccinated control animals and *n* vaccinated animals.

In the control group, we simulate single challenges of each animal with the *ID*_50_ by performing *n* Bernoulli trials with a probability of success of *p_c_* = 0.5. The probability of success corresponds to the probability with which an animal becomes infected after a single challenge. (By assuming the same probability *p_c_* for each animal, we ignore potential between-animal variation of the susceptibility to infection. This assumption will be relaxed below.) The results of these trials can be written as a vector ***x*** *_c_,* the entries of which were either zero (uninfected) or one (infected):



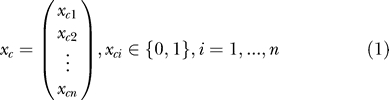



By summing over the elements of ***x*** *_c_,* we obtain the number of infected animals in the control group, *ι_c_*:



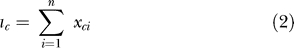



In the vaccinated group, we simulate single challenges with the *ID*_50_ similarly to the control group by performing Bernoulli trials. However, we assume that, because of vaccination, the probability of infection (or success) in the vaccinated group, *p_v_,* is lower than that in the control animals, *p_c_*. The relation of *p_v_* to the effect of the vaccine on the susceptibility of the host, *VE_S_,* is given by:



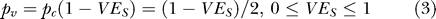



The results of these Bernoulli trials can again be written as a vector ***x*** *_v_,* and summing the elements of ***x*** *_v_* we obtain the number of infected animals in the vaccinated group, *ι_v_*.

The outcome of the simulated experiment can then be summarized in a contingency table as shown in Table 1. On this contingency table, we perform a standard one-tailed Fisher's exact test [24] to assess whether the fraction of infected animals in the vaccinated group is significantly lower than that in the control group.

**Table 1:**
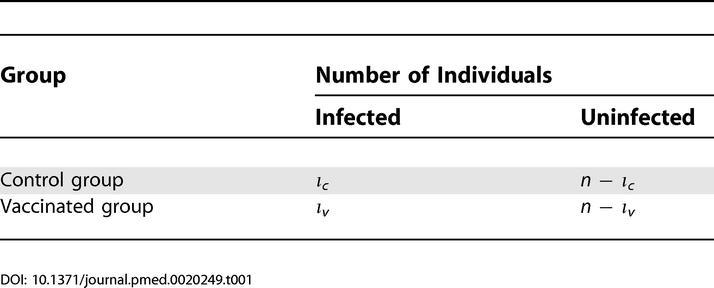
Contingency Table of a Single Low-Dose Challenge Experiment

### Simulation of Repeated Low-Dose Challenge Experiments

In our simulations of the repeated low-dose challenge experiments, we once more assume that we have *n* unvaccinated control animals and *n* vaccinated animals.

We again simulate challenges of each control animal with the *ID*_50_ by performing Bernoulli trials with a probability of success of *p_c_* = 0.5. Unlike in the simulations of the single low-dose challenge experiments, however, we now repeatedly challenge each animal until it is infected or until a maximum number of challenges, *C_max_,* has been performed. We assume that the probability of infection *p_c_* is independent of how often an animal has been challenged before. The results of these repeated Bernoulli trials can be written as two vectors, ***y*** *_c_,* which contains the number of challenges that have been performed on each animal:



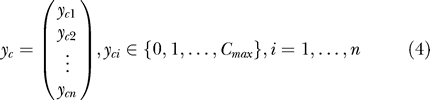



and ***s*** *_c_,* which contains information on whether a given animal is uninfected (zero) or infected (one):



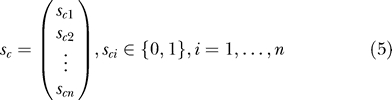



By summing over ***y*** *_c_,* we obtain the total number of challenges performed in the control group, η*_c_:*



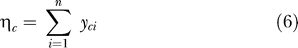



And, by summing over ***s*** *_c_,* we obtain the number of infected animals in the control group, *ι_c_:*



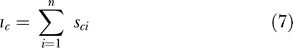



To simulate repeated low-dose challenges in the vaccinated group, we perform repeated Bernoulli trials with a probability of success *p_v_*. For a given vaccine efficacy *VE_S_,* *p_v_* is determined by equation 3. Analogously to the control group, the results of these repeated Bernoulli trials can be written as two vectors, ***y*** *_v_* and ***s*** *_v_,* and summing the elements of these two vectors yields the total number of challenges performed in the vaccinated group, η*_v_,* and, the number of infected animals in the vaccinated group, *ι_v_*.

As in the case of the single low-dose challenge design, the outcome of the simulated experiment can be summarized in a contingency table (Table 2). To assess whether the fraction of infected animals in the vaccinated group is significantly lower than that in the control group, we again perform a one-tailed Fisher's exact test [24]. In general, the number of challenges, η*_c_* and η*_v_,* is larger than the number of animals per group, *n*. This increase of numbers in the contingency table leads to increased statistical power of the repeated low-dose challenge design. To analyze the outcome of the simulated repeated low-dose challenge experiments, we chose to use Fisher's exact test rather than a more obvious Cox proportional hazards model because the latter depends on large sample asymptotics while we were interested in cases of small numbers of experimental animals.

**Table 2:**
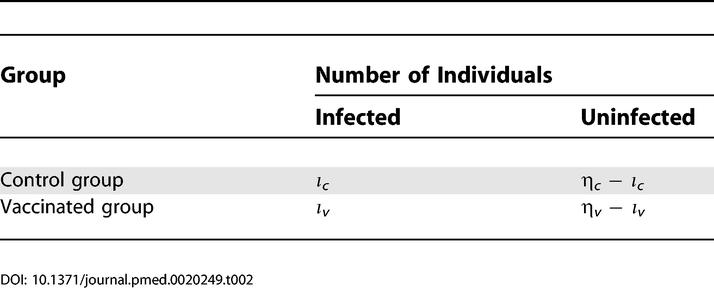
Contingency Table of a Repeated Low-Dose Challenge Experiment

### Heterogeneity in Infection Probabilities

In our mml:mathematical description of challenge experiments, we have assumed that animals within each group have equal infection probabilities—*p_c_* and *p_v_,* for the control and vaccinated groups, respectively. To simulate potential animal-to-animal variation in susceptibility to infection, we relaxed this assumption and assigned individual infection probabilities to each animal.

The individual infection probabilities are drawn from a β-distribution, which is often used as a prior distribution for binomial proportions. The β-distribution has two shape parameters, α and β. Its probability density is given by



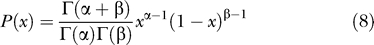



and its mean and variance are



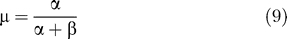





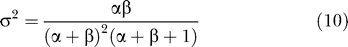



We assume that μ = *p_c_* in the control group and μ = *p_v_ =* (1 − *VE_S_*)*p_c_* in the vaccinated group. Further, we assume that the coefficients of variation, *CV =* σ/μ, of the distributions in the two groups are equal. With these assumptions, we can rewrite the two shape parameters of the β-distribution, α and β, in terms of the infection probability, *p,* and the coefficient of variation, *CV:*



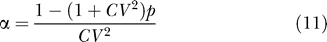





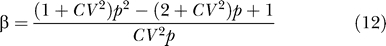



Hereby, *p = p_c_* for the control group and *p = p_v_* = (1 − *VE_S_*)*p_c_* for the vaccinated group.

To incorporate potential heterogeneity in susceptibility into the virtual low-dose challenge experiments, we replaced the probability of success in the Bernoulli trials (see above) with the individual infection probabilities.

### Power Analysis

To calculate the statistical power of the single and the repeated low-dose challenge designs, we performed 100,000 such simulated experiments for a given number, *n,* of animals per group. The statistical power can be estimated as the fraction of simulated experiments in which the vaccine is found to be significantly efficacious (significance level α = 0.05). We estimated the statistical power for the number of animals per group, *n,* ranging from one to 20, and for vaccine efficacies *VE_S_ =* 0.67, 0.8, and 0.9. The power analysis outlined above was implemented in the R Language of Statistical Computing [25]. An R-script that performs the power analysis presented here is provided as Protocol S1.

For large numbers of animals per group, *n,* the statistical power can be approximated using asymptotic theory. For the single low-dose challenge design the power is approximately (e.g., [26], p. 240):



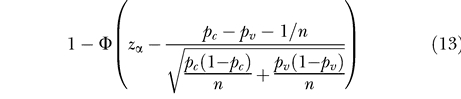



Hereby, Φ denotes the cumulative normal distribution,



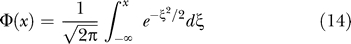



and *z*_α_ is the standard normal deviate associated with the one-tailed probability α (the significance level). Furthermore, *p_c_* and *p_v_* denote the infection probabilities of animals in the control and vaccinated groups, respectively, and *n* the number of animals per group. The term 1/*n* in the numerator is the continuity correction [27,28].

For the repeated low-dose challenge design, the number of challenges is not the same as the number of animals, *n,* but is a random variable. The number of challenges for each individual is geometrically distributed with a maximum of *C_max_*. The expected number of challenges in the control group, , and the vaccinated group, are



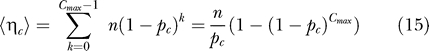



and



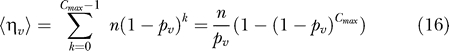



Substituting the expected number of challenges for the actual number, we can approximate the statistical power of the repeated low-dose challenge design as



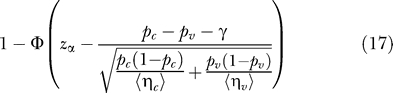



Hereby, γ = (1/〈η_*c*_〉 + 1/〈η_*v*_〉)/2 is the continuity correction. For *C_max_* = 1, equation 17 reduces to equation 13. Because the approximation in equation 17 involves the substitution of a random variable with its expectation, it is less accurate than the approximation for the power of the single low-dose challenge design in equation 13. The R-script provided as Protocol S1 also contains a function that calculates the statistical power using equation 17.

## Results

### Single Low-Dose Challenge Design Requires Large Numbers of Animals

How would we measure protection against infection in a low-dose challenge model? The most straight-forward design would involve a large number of hosts, some vaccinated and some unvaccinated. After challenge with a low dose, one would determine the fraction of infected hosts in vaccinated and unvaccinated groups, and assess whether there is a statistically significant difference in the fractions (see Figure 1A).

To assess how many animals would be required in a single low-dose challenge experiment, we performed a statistical power analysis (see Methods). The statistical power of an experimental design is defined as the probability that, in an experiment with an effective vaccine, the vaccine is correctly determined to be effective. Obviously the power depends on the efficacy of the vaccine (which is called the “effect size” in the context of power analysis) and the number of host animals used in the experiment. In the power analysis we performed, we assumed that we had equal numbers of unvaccinated and vaccinated animals, and that all animals within a group were equally susceptible to infection. Lastly, we assumed that the vaccine was “leaky” [29,30], i.e., that the susceptibility of vaccinated animals was by a constant factor lower than the susceptibility of the unvaccinated control animals.

In virtual experiments, we then challenged each (virtual) animal once with a challenge dose of one *ID*_50_, the dose at which on average 50% of the unvaccinated animals become infected after a single challenge. Using a one-sided Fisher's exact test, we tested whether the fraction of infected animals in the vaccinated group was significantly lower than in the control group. Performing 100,000 such virtual experiments for a given number *n* animals per group, we estimated the statistical power as the fraction of virtual experiments that yielded significant results (significance level α = 0.05).

**Figure 2:**
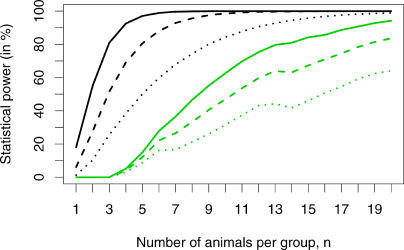
Power Analysis for the Repeated Low-Dose Challenge Design and the Single Low-Dose Challenge Design In our virtual experiments, we set the challenge dose equal to the *ID*_50_, and assumed that the vaccine efficacy was 67% (dotted lines), 80% (dashed lines), or 90% (solid lines). The graph shows the statistical power of the repeated low-dose challenge design (black lines) and the single low-dose challenge design (green lines) for a given number of animals per group as determined from 100,000 virtual experiments. If the vaccine is 90% effective, the statistical power of the repeated low-dose challenge design is higher than 95% with only five animals per group, as compared to only 15% for the single low-dose challenge design.

The result of this power analysis is shown by the green curves in Figure 2. We calculated the power for vaccine efficacies of 67%, 80%, and 90%. We found that, even for the highest vaccine efficacy of 90%, the single low-dose challenge design required more than 20 animals per group to reach a statistical power of 95%. Thus, the single low-dose challenge design is not feasible, or at least not practical, to assess the efficacy of a vaccine or other preventive interventions in animals.

### Repeated Low-Dose Challenge Design Does Not Require Large Numbers of Animals

We propose an alternative design involving repeated challenges of individual animals with low doses, which circumvents the disadvantage of the single low-dose challenge design that large numbers of host individuals are required. Repeated challenges effectively “recycle” host animals, thus increasing the statistical power of the experiment. In addition to increasing the statistical power of the experimental design, repeated challenges recapitulate much more realistically the circumstances of human exposure than single challenges. In this alternative design, the efficacy of a vaccine can be estimated by measuring the difference in the number of challenges needed to infect vaccinated versus unvaccinated hosts (see Figure 1B).

To show that this alternative design does not require unfeasibly large numbers of animals, we performed a statistical power analysis (see Methods). As for the single low-dose design, we assumed that we had equal numbers of unvaccinated and vaccinated animals, and that all animals within a group were equally susceptible to infection. We further made the important assumption that the susceptibility of an individual animal was independent of how often the animal was unsuccessfully challenged previously. This assumption is commonly adopted in statistical models that are used to estimate the transmission rate of HIV [14–17]. By making this assumption, we ignored that an unsuccessful challenge may induce some degree of immunity against subsequent challenges. We would like to emphasize, however, that this assumption is not crucial for our argument, unless the degree of induced immunity is very high. Lastly, we again assumed that the vaccine was leaky [29,30].

In virtual experiments, we then challenged the (virtual) animals repeatedly with a challenge dose of one *ID*_50_. We allowed for a maximum number of 20 challenges of each individual animal. Table 3 shows the outcome of one such virtual experiment. We analyzed the outcome of the virtual experiments with a one-tailed Fisher's exact test (see Methods). We again estimated the statistical power by performing 100,000 such virtual experiments for a given number *n* animals per group.

**Table 3:**
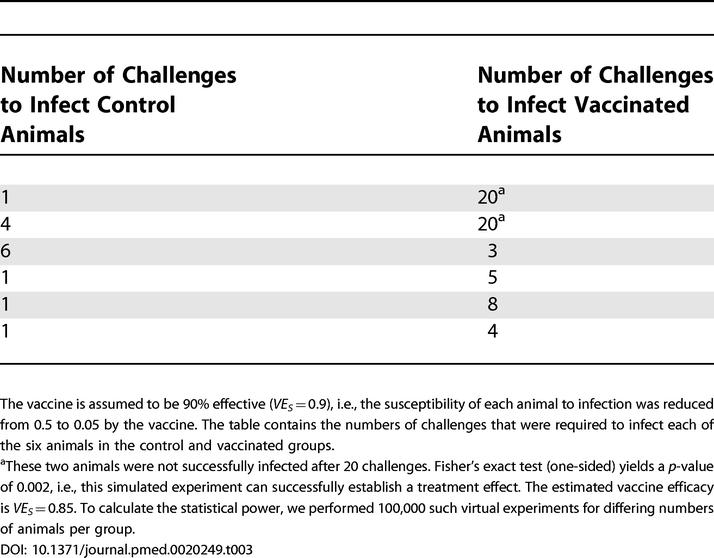
Outcome of One Virtual Repeated Low-Dose Challenge Experiment

Figure 2 shows the statistical power of the repeated low-dose challenge design as a function of the number of animals per group for varying vaccine efficacies (black lines), and compares it to the statistical power of the single low-dose challenge design (green lines). The statistical power achieved with the repeated low-dose challenge design is generally higher than that achieved with the single low-dose challenge design. If the vaccine is 90% effective (*VE_S_ =* 0.9), i.e., it reduces the susceptibility by a factor of ten, the number of animals per group could be as low as five to achieve more than 95% statistical power. In contrast, in single low-dose challenge experiments with the same number of animals per group the statistical power is only 15%. Thus, repeated low-dose challenge experiments are expected to require far fewer animals than single low-dose challenge experiments.

### How Often Should Virus Challenges Be Repeated?

**Figure 3:**
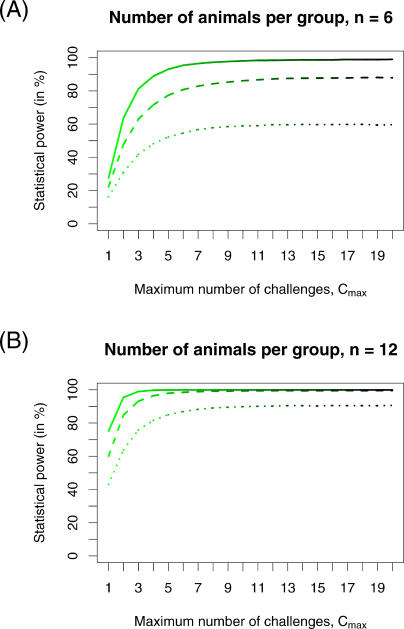
Impact of the Maximum Number of Challenges, *C_max_,* on the Statistical Power For this plot we assumed trials with vaccine efficacies of *VE_S_ =* 0.67 (dotted line), *VE_S_ =* 0.8 (dashed line), and *VE_S_ =* 0.9 (solid line). In (A) we calculated the statistical power for six animals per group, *n =* 6, and in (B) for 12 animals per group, *n =* 12.

To investigate how the maximum number of challenges affected the statistical power, we plotted the power against *C_max_* for trials involving six and 12 animals per group (Figure 3). We found that the power increases with *C_max_,* but for high *C_max_* the returns diminished considerably. The lower the number of animals per group, *n,* the higher the maximum number of challenges, *C_max_,* for which the power effectively saturated. Even for low numbers of animals per group, *n,* however, the maximum number of challenges, *C_max_,* needed to unfold the full potential of the repeated low-dose challenge design was in a feasible range.

### Impact of Animal-to-Animal Variation in Susceptibility

**Figure 4:**
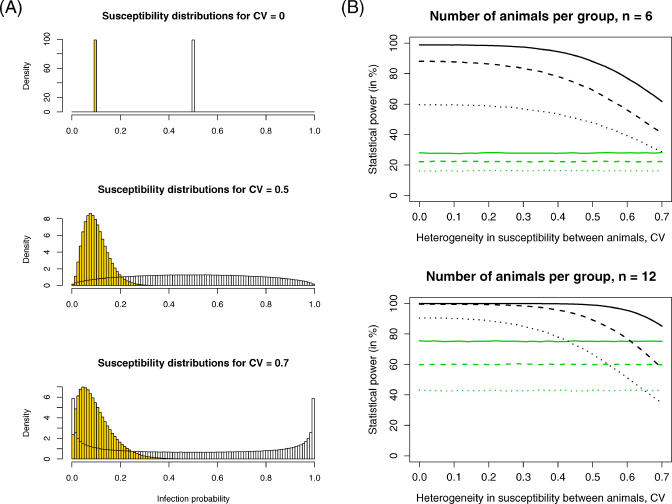
Impact of Heterogeneity in Susceptibility on the Statistical Power (A) Susceptibility distributions for different levels of heterogeneity, measured by the coefficient of variation, *CV,* of the susceptibility distribution. The vaccine is assumed to be 80% effective, *VE_S_ =* 0.8. (B) The statistical power depends on the coefficient of variation, *CV,* for the repeated low-dose challenge design (black lines) and the single low-dose challenge design (green lines). For these plots we assumed trials with six and 12 animals per group and vaccine efficacies of *VE_S_ =* 0.67 (dotted lines), *VE_S_ =* 0.8 (dashed lines), and *VE_S_ =* 0.9 (solid lines).

To study how potential heterogeneity in susceptibility affected the power of low-dose challenge trials, we simulated experiments in which each animal was assigned an individual infection probability (see Methods). In these simulations, the degree of heterogeneity was measured by the coefficient of variation, *CV,* of the susceptibility distributions. Figure 4A shows susceptibility distributions for three different values of *CV*.

We extended our power analysis by considering the impact of the heterogeneity parameter *CV* on the statistical power (Figure 4B). We found that the statistical power of the single low-dose challenge design was almost unaffected by animal-to-animal variation in infection probability, whereas, for the repeated low-dose challenge design, the power decreased with increasing heterogeneity. Importantly, however, the power did not decrease linearly with heterogeneity: it was sufficiently stable in the range 0 < *CV* < 0.3 and dropped mainly for *CV* > 0.3. Thus, over a wide range of potential animal-to-animal variation in susceptibility, low-dose challenge designs are sufficiently powered, and the power of the repeated low-dose experiments is superior to that of single low-dose challenge experiments.

## Discussion

Preclinical studies assessing the efficacy of potential vaccines, microbicides, or systemic chemoprophylaxis are usually conducted with very high virus challenge doses, which result in infection with certainty. Since these high challenge doses do not reflect the low probability of HIV transmission in humans, vaccines or prophylactic treatment strategies that are effective against “real life” exposures may go undetected in high-dose challenge experiments. For example, zidovudine was found to prevent a large fraction of perinatal HIV infections [23], even though studies in animal models, conducted with high challenge doses, could not establish any protection against infection by zidovudine [20–22].

In this paper, we investigated how efficacy trials of vaccines and preventive treatment could be conducted with low challenge doses in animal models. We showed that the repeated low-dose challenge design is expected to require far fewer experimental animals than commonly believed. It may therefore be feasible to conduct trials with low challenge doses, which more realistically simulate exposures of humans to HIV, allowing us to more directly and sensitively assess vaccine or treatment efficacy than with high-dose challenge experiments.

Owing to the concerns with high challenge doses, several research groups, including our own, have started to develop low-dose challenge models [31–34]. In these preliminary studies, infection could be achieved by challenging macaques intra-rectally [31], intra-vaginally [32,34], or orally [33].

Since adopting low-dose challenge approaches has far-reaching consequences for the design of efficacy trials of vaccines or preventive treatment in animal models, we would like to discuss how some important aspects of trial design, such as transient infections, the challenge schedule, the route of infection, and the phenotype and dose of the challenge strain, should be dealt with and could be optimized.

Using virus challenge doses that do not give rise to infection with certainty, one has to carefully define what one means by successful infection. This question is of particular importance in the repeated low-dose challenge design, because the efficacy of a preventive intervention is estimated on the basis of the number of challenges needed to infect an individual animal. Low-dose challenges have been observed to give rise to transiently detectable viremia [32–34]. Since transient infection is much more likely to lead to immunization [35], thus leading to lower probabilities of infection in subsequent challenges, we suggest considering transient viremia as successful infection and not to re-challenge animals that were transiently infected.

The time interval between challenges is also an essential parameter in the design of repeated low-dose challenge experiments. In the four ongoing repeated low-dose challenge studies [31–34], different approaches have been taken, with time intervals ranging from hours to a week. There may be logistical reasons for choosing short time intervals between challenges, but from a statistical standpoint, the time intervals should be large enough to allow the identification of the challenge that gives rise to infection. Otherwise, the statistical power of the experimental design will be suboptimal and a beneficial effect of the vaccine candidate may be missed.

In parallel to using more realistic, lower challenge doses, other crucial parameters of the experimental infection process, such as the route of transmission and the coreceptor usage of the challenge virus, should also be chosen to be as realistic as possible. Thus, we propose infecting intra-vaginally or intra-rectally in experiments that aim to assess a vaccine or prophylactic treatment against sexual transmission of HIV. Further, we suggest using challenge viruses that utilize CCR5 as coreceptor, such as for example SHIV-SF162P [36], rather than the standard strain SHIV89.6P, which has been found to use mainly CXCR4 [37,38]. These more realistic choices of the route of infection and coreceptor usage will permit the assessment of the efficacy of the preventive intervention in a setting that more accurately reflects HIV exposures of humans, and will enable us to carefully investigate the processes that give rise to infection.

The challenge dose in a low-dose challenge study is another parameter of crucial importance. Although the most realistic choice would be a challenge dose that gives rise to infection with a probability of approximately 0.0005–0.10 [14–17], such extremely low doses would require unfeasibly large numbers of repeated challenges per animal. Moreover, there is substantial variation in transmission rates due to differences in factors such as virus load or the presence of other infections of the genital tract [15–18], and theoretical studies suggest that preventing the transmission events that occur with higher probability would have a disproportionately large effect on controlling the epidemic [39]. To maximize their epidemiological relevance, low-dose challenge experiments should therefore involve challenge doses that reflect transmission probabilities at the upper end of the spectrum. As a compromise between the practicality of high doses and the sensitivity associated with realistically low doses, we propose the *ID*_50_. The *ID*_50_ can be estimated using well-established nonparametric methods like Spearman-Kärber [40] or single-parameter methods [41], and there is software available, such as a freely distributed package called ID50 developed by John Spouge (http://www.ncbi.nlm.nih.gov/CBBresearch/Spouge/Virology/, which allows an automated estimation of the *ID*_50_ from data generated in titration experiments.

The inability to detect sterilizing immunity in high-dose challenge experiments led to a shift of focus towards indirect effects of vaccine candidates on the pathogenicity of the infection and the infectiousness of the vaccinee. This shift of focus required the development of novel statistical models that allowed the estimation of these indirect effects [42,43]. Will the estimation of vaccine efficacy in repeated low-dose challenge studies also require the development of novel statistical techniques? The answer to this question depends on how much the realities of the infection process deviate from our idealized model. There are three potential deviations. First, we assumed in large parts of this study that the susceptibilities to infection were equal for all animals within each group. This is almost certainly not the case. Although we have shown that low-dose challenge experiments are sufficiently powered even if there is substantial animal-to-animal variation in susceptibility, we did not develop the statistical techniques that would allow the estimation of this variation. The extent of animal-to-animal variation in susceptibility can, in principle, be estimated, but this will probably require larger numbers of animals than the estimation of vaccine efficacy. Second, the vaccine may affect the susceptibility of individual animals differently. While we assumed in the present study that the vaccine is leaky, i.e., that the susceptibility is reduced by a constant factor in each animal, other modes of action of a vaccine are possible. In particular, some animals could be completely protected by vaccination, while others may remain completely susceptible. This mode of action is referred to as all-or-none [29,30]. Statistical methods based on maximum likelihood approaches exist that allow the determination of the mode of action of a given vaccine. However, these methods are based on large sample asymptotics, and exact methods will have to be developed to analyze the outcome of low-dose challenge experiments that involve small numbers of animals. Last, it will have to be determined whether the probability of infection changes with the number of challenges performed in a given animal, or, to put it differently, whether the animal has a “memory” of previous challenges. In our analysis, we assumed that the susceptibility of an animal did not change from challenge to challenge. If the probability of infection changes significantly with the number of challenges, however, the development of novel statistical models that take such changes into account will be necessary to adequately estimate vaccine efficacy.

In addition to the potential to assess the vaccine or microbicide efficacy more sensitively and in a more realistic setting, a low-dose challenge approach may enable us to answer questions that cannot even be asked in high-dose challenge models. Some of the most relevant of these questions relate to the effect of challenges that do not lead to infection. If a low-dose challenge does not give rise to infection, where was the virus blocked? Did the virus fail to establish an infection at all? Or did it replicate transiently, but was cleared by the host's immunity? And, very importantly, is an unsuccessfully challenged animal partially immunized against further challenges, or, alternatively, do unsuccessful challenges facilitate future infection by “seeding” animals with defective proviruses that may recombine with complementing viruses upon subsequent exposures [44]?

The answers to these questions would greatly enhance our understanding of HIV transmission and pathogenesis, and thus would provide further guidance toward an effective vaccine or microbicide. Furthermore, by assessing the protection against infection directly, we may be able to discern the specific types and levels of vaccine-induced cellular and humoral immune responses associated with sterilizing immunity [13]. This would provide important benchmarks by which to judge new vaccine candidates, and could also allow retrospective analysis of vaccine candidates evaluated earlier in high-dose challenge studies.

In conclusion, the repeated low-dose challenge approach may enable us to assess the potential efficacy of vaccines and prophylactic treatment strategies more realistically, and more sensitively than the standard high-dose challenge approach. The increased sensitivity may allow us to more rapidly identify interventions that significantly reduce the transmission of low-dose infections that characterize the natural spread of HIV.

## Supporting Information

Protocol S1 R-Function for the Calculation of the Statistical Power of Low-Dose Challenge Experiments (8 KB TXT).

**Patient Summary**

**Background**

Before trials of medicines or vaccines are done in humans, most are tested in animals. There are many controversies about these animal trials, including whether they mimic the human disease accurately. In testing vaccines for HIV, animals are mostly given high doses of the virus, whereas in real life people are often repeatedly exposed to small amounts of the virus. No vaccine that has been tested against HIV prevents infection in animals. It is possible that some of this lack of success may be due to the design of the vaccine trials rather than the vaccine itself.

**What Did the Authors Do?**

They wanted to look at experimental designs that allowed assessment of protection against infection with lower, and thus more realistic, doses of virus. Previously, researchers had suggested that many animals would be needed for this type of study. The authors wanted to see whether this was correct. They developed a model to test how well single and multiple low-dose experiments performed. They did this by simulating the experiments with doses of virus, assessing the results, and then repeating this procedure 100,000 times to estimate how valid a given experimental design was.

Their modeling showed that by repeatedly giving animals low doses of virus, it was possible to use a smaller number of animals than was needed for trials with a single low dose.

**What Do These Results Mean?**

It may be possible to use these results to plan trials of vaccines in animals that mimic more closely the way that humans are exposed to HIV, and hence the results may be more reliable for human disease.

**Where Can I Get More Information?**

MedlinePlus has a great deal of information on HIV:

http://www.nlm.nih.gov/medlineplus/aids.html

The Body has information targeted to both patients and health professionals:

http://www.thebody.com/index.shtml

## Abbreviations

*ID*50: infectious dose at which 50% of the animals become infected
